# New Insights on *Arabidopsis thaliana* Root Adaption to Ammonium Nutrition by the Use of a Quantitative Proteomic Approach

**DOI:** 10.3390/ijms20040814

**Published:** 2019-02-14

**Authors:** Inmaculada Coleto, Izargi Vega-Mas, Gaetan Glauser, María Begoña González-Moro, Daniel Marino, Idoia Ariz

**Affiliations:** 1 Department of Plant Biology and Ecology, University of the Basque Country (UPV/EHU), Apdo. 644, E-48080 Bilbao, Spain; inmaculada.coleto@ehu.eus (I.C.); izargiaida.vega@ehu.eus (I.V.-M.); mariabegona.gonzalez@ehu.eus (M.B.G.-M.); 2 Neuchâtel Platform of Analytical Chemistry, University of Neuchâtel, Avenue de Bellevaux 51, 2000 Neuchâtel, Switzerland; gaetan.glauser@unine.ch; 3 Ikerbasque, Basque Foundation for Science, E-48011 Bilbao, Spain; 4 Departamento de Biología Ambiental. Facultad de Ciencias, Universidad de Navarra, C/Irunlarrea 1, 31008 Pamplona, Spain

**Keywords:** ammonium, *Arabidopsis thaliana*, carbon metabolism, nitrogen metabolism, nitrate, proteomics, root, secondary metabolism

## Abstract

Nitrogen is an essential element for plant nutrition. Nitrate and ammonium are the two major inorganic nitrogen forms available for plant growth. Plant preference for one or the other form depends on the interplay between plant genetic background and environmental variables. Ammonium-based fertilization has been shown less environmentally harmful compared to nitrate fertilization, because of reducing, among others, nitrate leaching and nitrous oxide emissions. However, ammonium nutrition may become a stressful situation for a wide range of plant species when the ion is present at high concentrations. Although studied for long time, there is still an important lack of knowledge to explain plant tolerance or sensitivity towards ammonium nutrition. In this context, we performed a comparative proteomic study in roots of *Arabidopsis thaliana* plants grown under exclusive ammonium or nitrate supply. We identified and quantified 68 proteins with differential abundance between both conditions. These proteins revealed new potential important players on root response to ammonium nutrition, such as H^+^-consuming metabolic pathways to regulate pH homeostasis and specific secondary metabolic pathways like brassinosteroid and glucosinolate biosynthetic pathways.

## 1. Introduction

Nitrogen (N), despite being one of the essential macronutrients for plant development, is often a limiting element in agricultural soils. The two major inorganic N sources for plants in soils are nitrate (NO_3_^−^) and ammonium (NH_4_^+^). The first one is an anion (oxidation state of N, +5) and the second one, a cation (oxidation state of N, −3), thus, both N sources differ extremely in their chemical properties [1]. In the soil, both N sources are present. However, their relative abundance is reliant on its interaction with the microbiological and physicochemical characteristics of the soil, and plant preference for one or another form depends on the interplay between plant species and environmental variables such as soil properties or light [2]. The use of NO_3_^−^ as fertilizer, as well as the high nitrification rates commonly observed in agricultural soils when urea or NH_4_^+^ are applied, have made that crop species are mostly adapted to nitrate nutrition. However, the increasing use of ammonium-based fertilizers formulated with nitrification inhibitors, which have been proven useful to mitigate the effect of agriculture on the environment [3,4], demands a deeper study of plants N source preference. Ammonium nutrition may represent a stressful situation for a wide range of plant species when it is applied at high concentrations. Growth reduction is the most common symptom of ammonium stress [5,6]. The toxicity degree is dependent on genetic features (inter- and intraspecies) and on chemical traits, such as external NH_4_^+^ concentration and pH [7,8,9]. Indeed, pH is known to play a key role in plants response to ammonium nutrition; importantly, plants adapted to acidic conditions have sometimes been reported to be tolerant to ammonium stress [10,11].

NO_3_^−^, with net negative charge, is co-transported with two protons, whereas NH_4_^+^, with net positive charge, is mainly transported through electrogenic transport via ammonium transporters (AMTs) or cation channel [12,13]. This uptake difference in terms of charge balance can significantly influence the uptake of other mineral nutrients and also cell metabolic homeostasis [14,15]. Moreover, NO_3_^−^ and NH_4_^+^ assimilation is also different in terms of H^+^ balance, reductants consumption and redox balance [16]. For the synthesis of one glutamate molecule, NO_3_^−^ assimilation produces OH^-^ whereas NH_4_^+^ assimilation produces two H^+^ [17,18]. Thus, the balance of H^+^ production and consumption must be accurately controlled according to the N form absorbed to maintain the cytoplasmic pH and a favorable electrochemical gradient across cell membrane [19]. This control is exerted through the so-called “biophysical pH-stat” and “biochemical pH-stat”. The “biophysical pH-stat” is based on the buffering capacity of HPO_4_^2−^ and in the action of H^+^-pumps (e.g., ATPases). The “biochemical pH-stat” is based on the activation of H^+^-consuming metabolic pathways [20].

Although ammonium nutrition at high concentration (millimolar range, mM) is mostly reported as an unwanted situation that may affect crops yield, the metabolic adaptation to the presence of NH_4_^+^ as main N source, may entail benefits for plants. For instance, since ammonium nutrition is known to stimulate N assimilation machinery, an increase in protein yield has been reported in grains of wheat grown with NH_4_^+^ as N source [21]. Similarly, an increase in leaf glucosinolate content has been shown in ammonium-fed Arabidopsis and Brassica crops [15,22]. Besides, some works have reported a higher tolerance to abiotic stresses, such as drought and salinity, in different ammonium-fed plant species [23,24,25,26]. Moreover, considering the constant rising of atmospheric CO_2_ concentrations, some authors have argued that C3 plants growing under ammonium nutrition responded more positively to elevated CO_2_ than such plants growing under nitrate nutrition [1,27]. However, this is controversial since other works have not observed this effect [28,29].

Although plant response upon ammonium nutrition has been extensively studied, the molecular mechanisms governing the responses that lead plants to adapt their metabolism to tolerate this situation remain largely unknown. In this context, to find new actors, mechanisms and processes associated with plants ammonium response, we have performed a quantitative proteomic study in the root of Arabidopsis plants grown under a non-toxic ammonium condition, using nitrate nutrition as control. This approach provided some new clues for future research related to metabolic pathways and signaling processes involved in root adaptation to ammonium nutrition, such as the induction of secondary metabolism and the putative association between the gamma-aminobutyric acid (GABA) shunt, malate, and enzymes participating in biochemical pH-stat to regulate H^+^ balance.

## 2. Results and Discussion

### 2.1. Physiological Response of Arabidopsis Roots under Ammonium Nutrition

Most plant species are sensitive to long-term ammonium nutrition at high concentration [5,6]. In this work, despite the different N source supplied (nitrate vs. ammonium), plants showed similar total and root biomass (Table 1). Indeed, a relief from toxicity symptoms has often been observed when nutrient solutions are pH-buffered or when medium pH increases because this counteracts the medium acidification derived from ammonium nutrition [9,11,30]. Thus, growing the plant in a buffered medium (pH 6.5), we achieved a condition to study ammonium tolerance in Arabidopsis. Since plant root is the first organ that senses and responds to nutritional conditions, understanding how root adapts to non-toxic NH_4_^+^ nutrition is an important step to design practices for mineral N nutrition management in plants. In general, it is considered that the deleterious effect of ammonium nutrition at high concentrations in plants is a consequence of the excessive NH_4_^+^ uptake and accumulation in tissues, and plants with an enhanced synthesis of N-reduced compounds are more tolerant to ammonium nutrition [31,32]. To further ascertain whether ammonium-fed plants were suffering or not stress, we quantified internal NH_4_^+^ content, free amino acid and total soluble protein contents in roots. In this work, roots of ammonium-fed plants showed an increase of amino acid and soluble protein contents, whereas only a slight increase of internal NH_4_^+^ content compared with those grown with NO_3_^−^. These results indicate that plants were not facing a stressful situation (Table 1).

In order to identify root metabolic pathways differentially regulated in both N conditions that could be “targets” to further research in ammonium-fed plants, we performed a comparative proteomic analysis.

### 2.2. Overview of Proteomic Analysis in Arabidopsis Roots Grown under Exclusive Nitrate or Ammonium Supply

A quantitative proteomic analysis, with isobaric tags for relative and absolute quantitation (iTRAQ), was used to analyze relative abundance of proteins in four independent pools of Arabidopsis roots per treatment (1 pool = 120 individual plant roots). Peptides of six or more amino acids in length, and with a maximum of two missed cleavages were exclusively considered for the analyses. For protein quantification, only proteins identified in at least three out of four samples per treatment and with two or more unique peptides identified were considered. Following these criteria (detailed information in Materials and Methods section), we identified 4469 proteins and quantified 799, out of them 68 proteins were differentially abundant (*p* ≤ 0.05) in both N conditions (Table 2 and Supplementary Dataset S1). Among these 68 proteins, 31 showed a higher abundance in roots of ammonium-grown plants, whereas 37 showed a higher abundance in roots of nitrate-grown ones. Functional classification of differentially abundant proteins according to MapMan software analysis [33] revealed that a significant number of the differentially regulated proteins were associated with categories related to primary carbon (C) metabolism, in particular, to organic acid transformation, photorespiration, glycolysis, gluconeogenesis, carbohydrate metabolism, and amino acid metabolism (Table 2 and Supplementary Dataset S2). Importantly, most of these proteins showed higher abundance in root of ammonium-fed plants (Table 2). Differentially abundant proteins were also included in categories such as protein turnover (synthesis/degradation), signaling, abiotic stress, and redox response, among others. In addition, a number of proteins was related with transport processes, notably H^+^ transport, which is a key aspect when leading to pH homeostasis control under ammonium nutrition (Table 2). Despite the different H^+^ balance driven by distinct N forms used as N source, cytoplasmic pH stays mostly unchanged because of the pH-stat mechanisms [34]. These mechanisms for pH regulation are those related mainly to the biophysical pH-stat, mainly constituted by H^+^ pumps, H^+^ inclusion in vacuoles and H^+^ release in the rhizosphere, and biochemical pH-stat [34]. Curiously, in this study, two proteins related to H^+^ pumps were downregulated under ammonium nutrition, a P-type ATPase from the superfamily of cation-transporting ATPases (ATPase 2; P19456) and a V-type proton ATPase subunit E3 (P0CAN7) (Table 2). Consistent with these results, Marino et al. reported the lesser abundance of the proton pump-interactor 1 (O23144), which stimulates plasmatic membrane H^+^-ATPase activity in vitro conditions, in the leaves of Arabidopsis grown with ammonium as N source [15,35]. Furthermore, transcriptomic studies in ammonium-fed plants also showed downregulation of genes associated to H^+^ transport in vacuole and plasma membrane such as the vacuolar cation/proton exchanger 3-like gene (Solyc06g006110.2.1) in tomato, and the H^+^-transporting plasma membrane ATPases (AT3G60330; AT4G30190) in Arabidopsis [36,37]. In addition, sorghum roots exposed to ammonium concentrations above 1 mM also showed decreased H^+^-ATPase gene expression and activity [38]. It has been suggested that the biophysical pH-stat would be regulating pH homeostasis upon transitory pH variations; in contrast, it would not be effective upon long-term intracellular pH alterations [39]. Future studies about the relationship between ammonium uptake and homeostasis, and the “biophysical pH-stat” mechanisms, will be essential to further understand the potential involvement of H^+^ pumps and specifically ATPases on pH regulation associated with ammonium nutrition.

**Figure 1:**
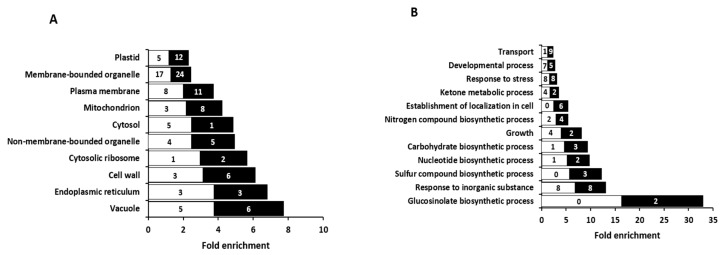
(**A**) Enriched categories for cellular component and (**B**) biological process of differentially abundant proteins in roots of *A. thaliana* plants cultured with ammonium (*p* ≤ 0.05). Number of proteins upregulated (white) and downregulated (black) by ammonium relative to nitrate, is indicated inside the bars.

Gene ontology (GO) enrichment analysis for cellular component (Figure 1A and Supplementary Dataset S3) and biological process (Figure 1B and Supplementary Dataset S3) was performed with BioMaps tool of VirtualPlant 1.3 [40]. Regarding cellular locations, almost every compartment was enriched, the vacuole being the cellular component showing the highest fold enrichment, followed by the endoplasmic reticulum and the cell wall (Figure 1A). Regarding biological processes, the GO enrichment analysis highlighted “glucosinolate biosynthetic process” as the category with the highest fold enrichment, followed by “response to inorganic substance” and “sulfur compound biosynthetic process” (Figure 1B).

### 2.3. Glucosinolate Biosynthesis is Modulated by Ammonium or Nitrate as N Source

As stated, the GO enrichment analysis highlighted the regulation of the glucosinolate (GLS) biosynthetic process by the N source provided (Figure 1b). GLS are abundant sulphur-containing secondary metabolites found almost exclusively in the Brassicaceae family, which are classified in function of their precursor amino acids. Indolic GLS are derived from Trp, aromatic GLS are derived from Phe or Tyr and aliphatic GLS are derived from Ala, Ile, Leu, Met, or Val. Arabidopsis Col-0 produces up to 40 different GLS that are mainly derived from Met and Trp [41]. The classical function of GLS is plant defense from insect and pathogen attack. Indeed, herbivore triggers GLS degradation and the generated degradation products are toxic for the pathogen [42]. Besides, although this aspect has been studied to a much lesser extent, GLS seem to be related with the response of Brassica plants to abiotic stresses such as salinity and water deficit [43,44]. Because GLS synthesis is linked with S and N metabolism, N availability can influence their accumulation in different Brassica crops [45,46]. Regarding the effect of ammonium nutrition, GLS synthesis induction has been reported in the leaves of Brassicaceae plants such as Arabidopsis, broccoli, and oilseed rape [15,22,47]. However, in the present study, the two differentially abundant proteins associated with “glucosinolate biosynthetic process”, identified and quantified in roots, were both downregulated in ammonium—relative to nitrate-fed roots; therefore, suggesting a different behavior of root tissue with respect to leaf tissue. These two downregulated proteins are 3-isopropylmalate dehydratase large subunit 1 (Q94AR8) and methylthioalkylmalate synthase 3 (Q9FN52), which are involved in side-chain methionine elongation, the precursor for aliphatic GLS biosynthesis (Table 2 and Supplementary Dataset 1). To assess whether the effect of N source on GLS metabolism proteins was also reflected in the content of GLS, individual GLS were quantified. In accordance with the downregulation observed in GLS-metabolic process, total GLS content was lower in ammonium-fed roots compared to nitrate-fed ones (Table 3). This decrease was mainly due to the contribution of aliphatic GLS; specifically, to glucohirsutin, 7-methylthioheptyl-GS and 8-methylthiooctyl-GS that were indeed the most abundant aliphatic GLS. Indolic GLS content was similar in both nutritional conditions and no aromatic GLS was detected (Table 3). Overall, it is clear that N source affects GLS synthesis; however, it remains to be elucidated how its differential regulation in shoots and roots takes place and whether GLS long-distance transport systems are involved in this organ-dependent regulation. This will be helpful for generating plants with increased GLS synthesis, which is desirable to promote natural plant defense and Brassica crops nutritional value. Indeed, GLS derivatives, in particular sulphoraphane, that is produced from glucoraphanin hydrolysis, have been associated with health-promoting activities [48].

### 2.4. Ammonium Nutrition and Secondary Metabolism in Arabidopsis Roots: Brassinosteroids and Hormonal Signaling Pathways

**Figure 2:**
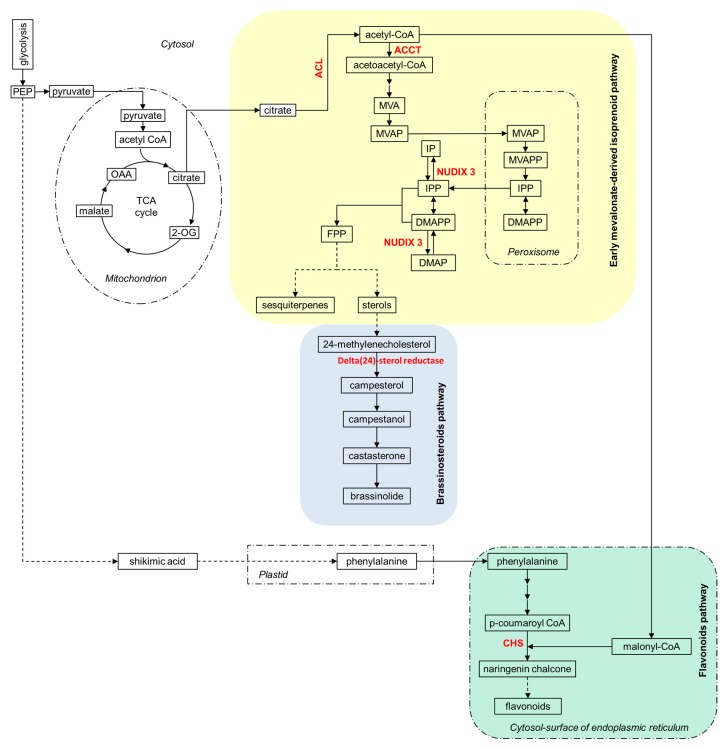
Induction of secondary metabolism in Arabidopsis roots by ammonium nutrition. Proteins with higher abundance in ammonium nutrition are highlighted in red bold text. Dotted arrows indicate non-detailed metabolic steps/transformations in a metabolic pathway. Abbreviations: 2-oxoglutarate (2-OG); acetyl-CoA acetyltransferase (ACCT); ATP-citrate lyase (ACL); chalcone synthase (CHS); dimethylallyl diphosphate (DMAPP); dimethylallyl phosphate (DMAP); farnesyl diphosphate (FPP); isopentenyl diphosphate (IPP); isopentenyl phosphate (IP); nudix hydrolase and a dipeptidyl peptidase III (NUDIX 3); mevalonate (MVA); oxalacetate (OAA); phosphoenolpyruvate (PEP); phosphomevalonate (MVAP).

Regulation of secondary metabolism has been reported in several species exposed to ammonium stress such as in tomato [36] or in Arabidopsis [49]. In the present study, quantitative proteomic analysis revealed that, besides glucosinolate biosynthesis, alternative secondary metabolic routes were also influenced by the N source. Ammonium-fed roots showed increased abundance of the ATP-citrate synthase alpha chain protein 3 (O80526), the subunit A of the heteromeric enzyme complex ATP-citrate lyase (ACL) in charge of acetyl-CoA synthesis (Figure 2). On one hand, acetyl-CoA is the central precursor of flavonoids and indeed a chalcone synthase (CHS; P13114) was more abundant in ammonium than in nitrate nutrition (Table 2 and Figure 2). On the other hand, acetyl-CoA can be condensed to acetoacetyl-CoA, by the action of acetoacetyl-CoA thiolase (AACT; Q854Y1-2), which also was more abundant in ammonium-fed roots. Acetoacetyl-CoA leads to the synthesis of early mevalonate-mediated isoprenoids [50] (Figure 2). AACTs are involved in Step 1 (1 of 3) of the sub-pathway that synthesizes mevalonate (MVA) [51]. Phosphomevalonate (MVAP), generated in cytosol by phosphorylation of MVA, enters peroxisome and after a couple of reactions, isopentenyl diphosphate (IPP) and its isomer, dimethylallyl diphosphate (DMAPP), the direct precursors of the entire class of isoprenoids derived from mevalonate, are produced [51]. IPP and DMAPP return to the cytosol and by the hydrolysis of the terminal phosphate bond, mediated by cytosolic phosphohydrolases such as Nudix hydrolase 3 (NUDX3; Q8L831), can be transformed into isopentenyl phosphate (IP) and dimethylallyl phosphate (DMAP), respectively [51,52]. NUDX3 was also more abundant in roots of ammonium-fed plants with respect to nitrate-fed ones (Figure 2). Thus, the increased abundance of these three proteins in ammonium-fed roots, participating in the early mevalonate-mediated isoprenoid biosynthesis pathway, suggests that this metabolic route may be induced and strongly modulated at such nutrition conditions. This pathway may lead to the synthesis of brassinosteroids (BRs). Indeed, the protein delta(24)-sterol reductase (Q39085), involved in the conversion of the early BR precursor 24-methylenecholesterol to campesterol, also showed increased abundance in ammonium nutrition (Table 2 and Figure 2). Interestingly, BRs have been recently related to the regulation of the AMT1-type ammonium transport proteins in Arabidopsis and rice [53,54]. In rice, BRs induce the gene expression of OsAMT1;1 and OsAMT1;2 ammonium transporters [54]. Furthermore, the authors identified ABI3/VP1-Like 1 (RAVL1), a regulator of BRs homeostasis, as a direct regulator of OsAMT1;2, overall showing an important link between BRs and the transcriptional regulation of ammonium uptake [54]. In contrast, in Arabidopsis, it appears that BRs will be acting as negative regulators of AMT1 transporters [53]. Overall, it seems that BR-mediated regulatory circuits are somehow connected with ammonium uptake and signaling in a species-dependent manner. Future works will be essential to shed further light on the complex interaction between hormonal signaling pathways and nutrient uptake, notably in the context of BRs–ammonium relationship.

### 2.5. C/N Metabolism Modulation in Ammonium-Fed Plants May Be Driven by Alternative C Provision Routes to Tricarboxylic Acid (TCA) Cycle while Contributing to H^+^ Balance

**Figure 3:**
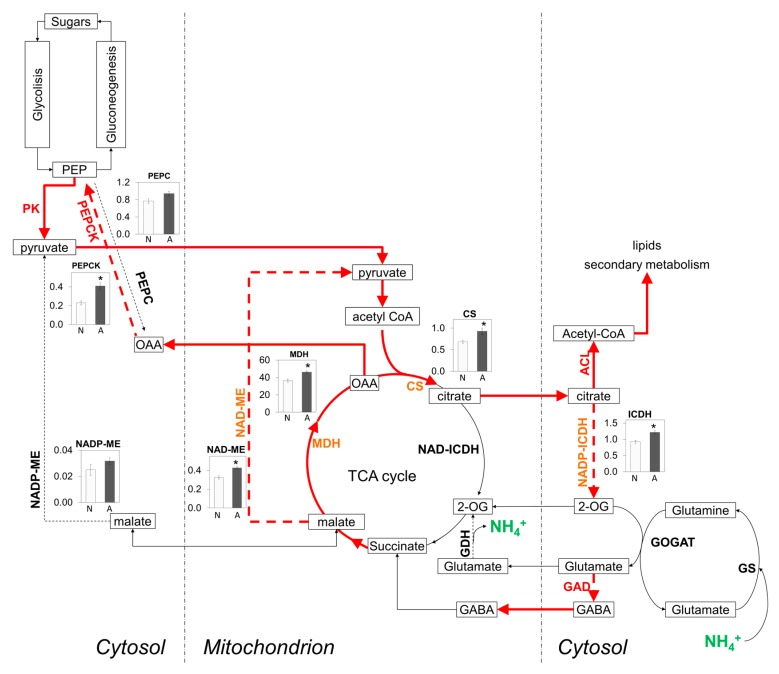
C anaplerotic routes (dotted arrows) in ammonium-fed roots of *A. thaliana* plants. Ammonium (NH_4_^+^) release or incorporation to metabolic pathways is highlighted in green bold text. Induced routes and proteins with higher abundance in ammonium relative to nitrate nutrition are highlighted in red (bold lines and text). Increased activity of anaplerotic and TCA-cycle enzymes are shown in orange bold text and emphasized with an asterisk (*) in the activity graphs. Graphs represent enzyme activity of Arabidopsis roots fed with ammonium (grey bars) or with nitrate (white bars). Enzyme activities are expressed as: CS (nmol CoA mg^−1^ FW min^−1^); ICDH (nmol NADP mg^−1^ FW min^−1^); MDH (nmol NADP mg^−1^ FW min^−1^); NAD-ME (nmol NAD mg^−1^ FW min^−1^); NADP-ME (nmol NADP mg^−1^ FW min^−1^); PEPC (nmol NADH mg^−1^ FW min^−1^); PEPCK (nmol NADH mg^−1^ FW min^−1^). Data shown in graphics represent mean values ± SE (n = 4). Asterisk (*) indicates significant N source effect (*t*-test, *p* < 0.05). Abbreviations: ATP-citrate lyase (ACL); citrate synthase (CS); gamma-amniobutyric acid (GABA). glutamate decarboxylase (GAD); glutamate dehydrogenase (GDH); glutamate synthase (GOGAT); glutamine synthetase (GS); NAD-isocitrate dehydrogenase (NAD-ICDH); NADP-isocitrate dehydrogenase (NADP-ICDH); malate dehydrogenase (MDH); NADP-malic enzyme (NADP-ME); NAD-malic enzyme (NAD-ME); phosphoenolpyruvate (PEP); phosphoenolpyruvate carboxylase (PEPC); phosphoenolpyruvate carboxykinase (PEPCK); pyruvate kinase (PK).

The C/N balance in plants is regulated by the availability of C skeletons, energy, and reductants for the N assimilatory pathways [55]. One of the known consequences of ammonium nutrition is the induction of ammonium assimilation machinery, which demands high energy and carbon consumption. In this study, several proteins associated with C metabolism were more abundant in roots of Arabidopsis plants grown under a non-toxic ammonium condition (Table 2 and Figure 3). In this line, previous studies reported that ammonium accumulation in roots triggering ammonium toxicity may be partially mitigated by the provision of extra C [29,56]. Interestingly, supplementary C would be not only serving N assimilation but also improving cell ion balance and managing respiration rates and ATP availability [56]. Indeed, although the respiratory cost of ammonium assimilation is not as much of that of nitrate, the overall effects of ammonium stress have been associated with the increased capacity of respiratory bypass pathways [57,58]. Specifically, the capacity of alternative oxidase (AOX) is substantially elevated in plants grown on ammonium [57,59]. AOX together with pyruvate kinase (PK; Q9FM97), protein more abundant in ammonium-fed roots of this study (Table 2 and Figure 3), have been described as a H^+^-sink unit of the revised biochemical pH-stat mechanism under aerobic conditions [60,61]. This is a key aspect, since the control of pH homeostasis is critical for the plant to face ammonium stress [9,30,62].

As previously mentioned, this proteomic study also revealed a number of proteins associated with secondary metabolism (Table 2 and Figure 2) and several authors have associated the production of secondary metabolites with cytoplasmic acidification [61,63]. Furthermore, Sakano suggested that AOX activation may also be deeply involved in the oxidation of excess reducing equivalents produced during the synthesis of secondary metabolites [61].

Besides AOX and PK, this study also showed increased abundance of other C metabolism-related proteins whose enzymatic activity consumes H^+^ such as phosphoenolpyruvate carboxykinase, (PEPCK; Q9T074) and glutamate decarboxylase (GAD; Q42472; Figure 3). We determined the enzyme activity of PEPCK and, in agreement with the proteomics results, it was also significantly higher in ammonium-fed plants (Figure 3). Malate dehydrogenase activity, which converts malate to the PEPCK substrate, oxaloacetate (OAA), was also increased under ammonium nutrition (Figure 3). The role of PEPCK in the metabolism of ammonium-fed plants regulating pH, by consuming H^+^ via malate decarboxylation to pyruvate by the sequential action of MDH, PEPCK, and PK has been previously suggested, notably, in the more active tissues in the N metabolism, such as the pericycle [18,64,65]. Furthermore, PEPCK abundance and activity also increased in cucumber plants exposed to ammonium and acidification conditions [66].

In roots of plants grown under ammonium nutrition, TCA cycle usually functions in an open-mode, since almost all the 2-oxoglutarate (2-OG) generated is diverted into amino acids synthesis [67]. Thus, to replenish pyruvate pool to ensure the supply of 2-OG, the anaplerotic pathways associated to TCA cycle have been suggested to bear a predominant role. Importantly, MDH and PEPCK, apart from their role in the “biochemical pH-stat”, are a part of these anaplerotic pathways together with malic enzyme (ME) and phosphoenolpyruvate carboxylase (PEPC). Overall, these anaplerotic routes were enhanced in ammonium-fed roots compared to the nitrate-fed ones (Figure 3).

The increased 2OG production by isocitrate dehydrogenase (ICDH) (Figure 3) may induce Glu production and its derivate amino acids (Table 1). For instance, GAD, in charge of GABA synthesis and whose abundance is increased in ammonium-fed plants (Table 2 and Figure 3) can be induced and stimulated by increases in cytosolic Ca^2+^ (via Ca^2+^/CaM) or H^+^ concentrations and thus, it has also been related to cell pH regulation [19,68]. GABA concentration is influenced by different environmental changes, inter alia, N form. Indeed, GABA content has already been shown to increase in ammonium-grown Arabidopsis plants relative to nitrate-fed plants [69]. Additionally, GABA and malate appear to be tightly connected and to participate, among others, in TCA cycle regulation and in the regulation of electrical potential across membranes acting on aluminum-activated anion transporters (AMLTs) [70,71]. Thus, a proper C and N metabolic adaptation in roots coordinated with NH_4_^+^ uptake, transport and storage appears essential in order to maintain cell pH, reductant, and electrochemical homeostasis upon plants growth under ammonium nutrition.

Finally, the present study provides new hints of metabolic pathways and signaling processes that can be involved in root adaptation to ammonium nutrition, a process that although thoroughly studied continues being still poorly understood. Among the novel points that arise from this study, the connection between ammonium nutrition and secondary metabolism and the putative association between GABA shunt and TCA cycle associated enzymes to regulate H^+^ balance and plasma membrane electrical potential deserve special attention in future research.

## 3. Materials and Methods

### 3.1. Plant Culture and Experimental Design

Plants used in this study were cultured as described in [15]. Briefly, seeds of *Arabidopsis thaliana* ecotype Col-0 were sterilized and cultured with a modified Murashige and Skoog solution (2.25 mM CaCl_2_, 1.25 mM KH_2_PO_4_, 0.75 mM MgSO_4_, 5 mM KCl, 0.085 mM Na_2_EDTA, 5 µM KI, 0.1 µM CuSO_4_, 100 µM MnSO_4_, 100 µM H_3_BO_3_, 0.1 µM CoCl_2_, 100 µM FeSO_4_, 30 µM ZnSO_4_, and 0.1 µM Na_2_MoO_4_) supplemented with 0.5% sucrose and 20.5 mM MES (pH 6.5) [72]. Nitrogen source was added at a concentration of 2 mM as 1 mM (NH_4_)_2_SO_4_ for ammonium-based nutrition or 1 mM Ca(NO_3_)_2_ for nitrate nutrition. To equilibrate the Ca^2+^ supplied together with the NO_3_^−^, NH_4_^+^-fed plants were supplemented with 1 mM CaSO_4_. The experimental design of this study, in terms of N concentration, pH, volume, and renew frequency of nutrient solution, was selected according to results obtained in previous nutritional studies with Arabidopsis plants [9,15].

Plants were stratified at 4 °C for four days in the dark and then moved into a growth chamber under the following controlled conditions: 14 h, 200 µmol·m^−2^·s^−1^ light intensity, 60% relative humidity, and 22 °C (day conditions); 10 h, 70% relative humidity, and 18 °C (night conditions).

First, plants were germinated and cultured during 9 days in 0.6% agar Petri dishes with the nutrient solution described above. After this time, seedlings were transferred to sterile 24-well plates containing 1 mL of the same nutrient solution used for seed germination without agar (one plant per well). Then, plates were kept under continuous shaking (120 rpm) for 12 additional days and the liquid nutrient solution was renewed on days 5 and 9. Four independent experiments were carried out, each one with 10, 24-well plates. Each plate contained 12 plants per treatment. When harvesting, shoots and roots of plants within each plate and treatment were pooled separately, dried with paper towels and the biomass was recorded. For proteomic and metabolic analysis, all the roots within each experiment and treatment (120 plants) were pooled together, immediately frozen in liquid nitrogen and stored at −80 °C.

### 3.2. Ammonium and Total Free Amino Acid Quantification

Root extracts for ammonium and total free amino acids quantification were obtained by adding 20 µL of ultrapure water per milligram of tissue. The homogenates were incubated at 80 °C during 5 min and centrifuged at 16,000*g* and 4 °C for 20 min and then, supernatants were recovered.

Ammonium content was determined following the phenol hypochlorite method [73]. For total free amino acids quantification, the ninhydrin method was followed using glutamine as a standard for the calibration curve [74].

### 3.3. Soluble Protein Quantification and Enzyme Activities Determination

For soluble protein and enzyme activities determination, frozen root powder was homogenized with extraction buffer (10 µL per milligram of tissue). Extraction buffer was composed by 0.1% Triton X-100, 10% glycerol, 0.5% polyvinylpolypyrrolidone, 50 mM HEPES pH 7.5, 10 mM MgCl_2_, 1 mM EDTA, 1 mM EGTA, 10 mM dithiothreitol, 1 mM phenylmethylsulfonyl fluoride, 1 mM ε-aminocaproic acid and 10 µM leupeptin. Homogenates were then centrifuged at 16,000*g* for 20 min at 4 °C and the supernatants recovered. Soluble protein content was determined by a dye binding protein assay (Bio-Rad Bradford Protein assay) using BSA as a standard for the calibration curve. Phosphoenolpyruvate carboxylase (PEPC), NAD-dependent malic (NAD-ME), NADP-dependent isocitrate dehydrogenase (ICDH), and malate dehydrogenase (MDH) were assayed as described in [9]. The following reaction buffers were used: for PEPC activity assay (100 mM Tricine-KOH (pH 8), 5 mM MgCl_2_, 5 mM NaF, 0.25 mM NADH, 6.4 U of malate dehydrogenase mL^−1^, 2 mM NaHCO_3_ and 3 mM phosphoenolpyruvate); for NAD-ME activity assay (50 mM HEPES-KOH (pH 8), 0.2 mM EDTA-Na_2_, 5 mM DTT, 2 mM NAD, 5 mM malate, 25 μM NADH, 0.1 mM Acetyl CoA and 4 mM MnCl_2_); for NADP-dependent malic enzyme (NADP-ME) activity assay (100 mM Tris-HCl (pH 7), 10 mM MgCl_2_, 0.5 mM NADP and 10 mM malate); for ICDH assay (100 mM Tricine-KOH (pH 8), 0.25 mM NADP, 5 mM MgCl_2_ and 5 mM isocitrate); for MDH assay (100 mM HEPES-KOH (pH 7.5), 5 mM MgSO_4_, 0.2 mM NADH and 2 mM oxaloacetate). Phosphoenolpyruvate carboxykinase (PEPCK) was assayed using a reaction buffer composed by 100 mM HEPES-KOH (pH 6.8), 25 mM DTT, 100 mM KCl, 90 mM KHCO_3_^−^, 1 mM ADP, 6 mM MnCl_2_, 0.2 mM NADH, 7 U of malate dehydrogenase mL^−1^ and 6 mM phosphoenolpyruvate, as described in [75]. Enzyme activities were assayed by spectrophotometry at 340 nm, monitoring evolution (formation or extinction) of NAD(P)H at 30 °C for 20 min. In the case of MDH, 10-fold diluted extracts were used. In citrate synthase (CS) activity assay, the extraction buffer was the same as that described above, except for DTT, which was not added. Protein extracts were incubated at 30 °C for 20 min with a reaction buffer (100 mM Tris-HCl (pH 8), 1 mM oxaloacetate, 0.25 mM acetyl coenzyme A and 0.1 mM 5,5′-dithiobis (2-nitrobenzoic acid), DTNB). CS activity was measured by spectrophotometry at 412nm, monitoring the absorbance originated by the formation of 2-nitro-5-thiobenzoic acid (TNB) [76]. All reagents were purchased from Sigma-Aldrich (St. Louis, MO, USA).

### 3.4. Glucosinolate Determination

For glucosinolate determination around 25 mg of frozen root powder were extracted by adding 1 mL of MeOH:water (70:30). The mixtures were homogenized in a Tissue Lyser (Retsch MM 400, Haan, Germany) and incubated for 15 min at 80 °C to inactivate myrosinase. Then, homogenates were centrifuged for 20 min at 16,000*g*. Glucosinolates were determined from supernatants by ultra-high performance liquid chromatography-quadrupole time-of-flight mass spectrometry (UHPLC/Q-TOF-MS) analyses using an Acquity UPLC from Waters (Milford, MA, USA) interfaced to a Synapt G2 QTOF from Waters (Milford, MA, USA)with electrospray ionization as described previously [77]. Glucosinolates were quantified using glucoraphanin and glucobrassicin as standards (Phytolab, Vestenbergsgreuth, Germany.

### 3.5. Proteomic Analysis

#### 3.5.1. Sample Preparation and Labeling for Proteomic Analysis

Proteins were extracted from 50 mg of root fresh weight (FW) homogenized in 0.5 mL of an extraction buffer composed by 7 M urea, 2 M thiourea, 4% CHAPS, 2% Triton X-100, 50 mM DTT, and 0.5% plant protease inhibitor and phosphatase inhibitors cocktails (Sigma-Aldrich, St. Louis, MO, USA). Then, homogenates were centrifuged for 15 min at 10,000*g* and 4 °C and total protein precipitated from 200 µL of supernatant with methanol and chloroform (600 µL methanol, 15 µL chloroform, and 450 µL ultrapure water). Mixtures were spun (in a vortex) and centrifuged for 1 min at 14,000*g*. The aqueous phase was then removed, an additional 450 µL of methanol added, and the centrifugation step was repeated. After discarding the methanol phase, protein pellets were dried in a vacuum centrifuge and resuspended into 7 M urea, 2 M thiourea, and 4% CHAPS. Global experiments were carried out with four independent biological samples in each experimental condition. Each sample corresponded to a pool of 120 plants. Protein extracts (150 μg) were precipitated with methanol/choloroform, and pellets dissolved in 7 M urea, 2 M thiourea, 4% (*v*/*v*) CHAPS. Protein was quantified with the Bradford assay kit (Bio-Rad, Hercules, CA; USA). A shotgun comparative proteomic analysis of total root extracts using an iTRAQ 8-plex experiment was performed [78]. iTRAQ labeling of each sample was made according to the manufacturer’s protocol (Sciex, Framingham, MA, USA). Total protein (100 μg) from each sample was reduced with 50 mM tris (2-carboxyethyl) phosphine (TCEP) at 60 °C for 1 h. Cysteine residues were alkylated with 200 mM methylmethanethiosulfonate (MMTS) at room temperature for 15 min. Trypsin (Promega, Fitchburg, WI, USA), 1:20, *w*/*w*, was used for protein enzymatic cleavage at 37 °C for 16 h. Each root tryptic digest was labeled by incubation (1 h) according to the manufacturer’s instructions with one isobaric amine-reactive tags, as follows: Tag113, ammonium media-1; Tag114, ammonium media-2; Tag115, ammonium media-3; Tag116, ammonium media-4; Tag117, nitrate media-1; Tag118, nitrate media-2; Tag119, nitrate media-3; Tag121, nitrate media-4. Then, every set of labeled samples was independently pooled and evaporated until <40 μL by vacuum centrifugation. Unless otherwise stated all reagents were purchased from Sigma-Aldrich (St. Louis, MO, USA).

#### 3.5.2. Peptide Fractionation

The peptide pool was injected to an Ettan LC system with a X-Terra RP18 pre-column (2.1 × 20 mm) and a high pH stable X-Terra RP18 column (C18; 2.1 mm × 150 mm; 3.5 μm) (Waters, Milford, MA, USA) at a flow rate of 40 μL/min, increasing in this way the proteome coverage. Elution of peptides was made with a mobile phase B of 5–65% linear gradient over 35 min (A, 5 mM ammonium bicarbonate in water at pH 9.8; B, 5 mM ammonium bicarbonate in acetonitrile at pH 9.8). Eleven fractions were collected, evaporated under vacuum and reconstituted into 20 μL of 2% acetonitrile, 0.1% formic acid, 98% MilliQ water previous to mass spectrometric analysis.

#### 3.5.3. Triple-TOF 5600 Mass Spectrometry (MS) Analysis

The split of peptides was made by reverse phase chromatography using an Eksigent nanoLC ultra 2D pump fitted with a 75 μm ID column (Eksigent 0.075 mm × 25 cm). Samples were desalted and concentrated with a 0.5 cm length 300 μm ID pre-column, which was packed with the same chemistry as the separating column. Mobile phases were 100% water 0.1% formic acid (buffer A) and 100% Acetonitrile, 0.1% formic acid (buffer B). The column gradient (237 min) used was a two-step gradient, first from 5% B to 25% B in 180 min and second, from 25% B to 40% B in 30 min. Column was equilibrated in 95% B for 10 min and 5% B for 15 min. Along the entire process, the pre-column was in line with column, and flow during the gradient was maintained at 300 nL/min. The separated peptides eluted from the column were analyzed using an AB Sciex 5600 TripleTOF™ system (Sciex, Framingham, MA, USA). Information data was acquired upon a survey scan (mass range from 350 *m*/*z* up to 1250 *m*/*z*; scan time: 250 ms). Top 35 peaks were selected for fragmentation. Minimum accumulation time for MS/MS was set to 100 ms (3.8 s of total cycle time). Product ions were scanned in a mass range from 100 *m*/*z* up to 1700 *m*/*z* and excluded for further fragmentation during 15 s.

#### 3.5.4. Data Analysis

Analyses of raw data (.wiff, Sciex) were performed with MaxQuant software [79]. Peak list was generated with the default Sciex Q-TOF instrument parameters except for the main search peptide tolerance that was set to 0.01 Da, and the MS/MS match tolerance that was increased up to 50 ppm. Minimum peptide length was set to six amino acids. Two databases were used. A contaminant database (.fasta) was first used to filter out contaminants. Peak lists were searched against the TAIR10 *A*. *thaliana* database (www. arabidopsis.org), and Andromeda was used as a search engine [80]. The search parameters allowed for methionine oxidation and cysteine modification by MMTS. Reporter ion intensities were bias corrected for the overlapping isotope contributions from the iTRAQ tags according to the certificate of analysis provided by the reagent manufacturer (Sciex, Framingham, MA, USA). The maximum false discovery rates (FDR) were set to 1% at the protein and peptide levels. Analyses were limited to peptides of six or more amino acids in length, and considering a maximum of two missed cleavages. Proteins identified by site (identification based only on a modification), reverse proteins (identified by decoy database) and potential contaminants were filtered out. Only proteins with more than one missing value was accepted (i.e., protein identified in three out of the four replicates) and was rescued by replacing it with the mean of the rest of the in-group samples. Data were normalized and transformed for later comparison using quantile normalization and log2 transformation, respectively. The Limma Bioconductor software package in R was used for ANOVA analyses. Significant and differential data were selected by a *p* value < 0.05.

#### 3.5.5. Functional Classification and Gene Ontology Enrichment Analysis

Functional classification of the differentially abundant proteins was carried out according to MapMan software (http://mapman.gabipd.org/es/mapman, version 3.6.0) [33]. Gene ontology (GO) enrichment analysis and visualization for cellular component and biological process were performed with BioMaps tool of VirtualPlant 1.3 using the *A. thaliana* Columbia tair10 genome as background population [40]. Over-representation was calculated with Fisher’s exact test, with a cut-off value of *p* ≤ 0.05.

### 3.6. Statistical Analyses

Proteomics data statistical analysis is described in the above section.

For biomass and metabolic data, statistical analysis was carried out using IBM SPSS 22.0 software (IBM Corp., Armonk, NY, USA). The significance of the results was assessed using independent samples Student *t*-test with a *p* value < 0.05.

## Figures and Tables

**Table 1:** Biomass, and NH_4_^+^, amino acids and soluble protein contents in 21 days-grown *Arabidopsis thaliana* plants (9 days in agar plates plus 12 days in 24-well plates) with nitrate or ammonium as sole N source.

Parameter	Nitrate	Ammonium
Total plant biomass (mg·FW·plant^−1^)	24.88 ± 1.48	24.41 ± 2.79
Shoot biomass (mg·FW·plant^−1^)	16.61 ± 0.69	**14.35 ± 0.31**
Root biomass (mg·FW·plant^−1^)	8.27 ± 0.82	10.06 ± 1.89
Root NH_4_^+^ content (nmol·mg^−1^ FW)	0.41 ± 0.03	**0.57 ± 0.05**
Root total free amino acids (nmol·Gln·mg^−1^·FW)	4.02 ± 1.5	**11.57 ± 0.89**
Root total soluble protein (µg·mg^−1^·FW)	4.1 ± 0.39	**9.13 ± 1.19**

Values represent mean ± SE (*n* = 20, for biomass and *n* = 4, for ammonium, amino acids and protein values). Significant differences between treatments are highlighted in bold text (Student *t*-test; *p* < 0.05). FW: Fresh weight.

**Table 2:** Functional classification of proteins showing differential abundance in roots of nitrate- and ammonium-cultured *A. thaliana* plants.

Protein Description	TAIR ID	Uniprot ID	*p*-Value	Fold Change NO_3_^−^/NH_4_^+^
**Organic acid transformation**				
Dihydrolipoyllysine-residue acetyltransferase component 5 of pyruvate dehydrogenase complex	AT1G34430	Q9C8P0	0.010	5.27
Dihydrolipoyl dehydrogenase 1	AT1G48030	Q9M5K3	0.019	0.30
ATP-citrate synthase alpha chain protein 3	AT1G09430	O80526	0.001	0.11
**Photorespiration**				
Serine hydroxymethyltransferase 3, chloroplastic	AT4G32520	Q94JQ3	0.011	0.19
**Carbohydrate metabolism**				
ADP-glucose pyrophosphorylase family protein	AT1G74910	F4HXD1	0.008	0.13
**Glycolysis**				
Pyruvate kinase	AT5G56350	Q9FM97	0.009	0.21
**Gluconeogenese/Glyoxylate cycle**				
Phosphoenolpyruvate carboxykinase (ATP)	AT4G37870	Q9T074	0.010	0.14
**Mitochondrial electron transport**				
Gamma carbonic anhydrase-like 1	AT5G63510	F4KAG8	0.003	0.17
**Amino acid metabolism**				
Glutamate decarboxylase 2	AT1G65960	Q42472	0.027	0.24
Aspartate semialdehyde dehydrogenase	AT1G14810	Q8VYI4	0.040	5.29
Methylmalonate-semialdehyde dehydrogenase (acylating)	AT2G14170	A8MQR6	0.048	4.36
**Secondary metabolism**				
3-isopropylmalate dehydratase large subunit 1	AT4G13430	Q94AR8	0.003	5.48
Methylthioalkylmalate synthase 3	AT5G23020	Q9FN52	0.003	3.25
Acetyl-CoA acetyltransferase	AT5G48230	Q854Y1-2	0.049	0.30
Betaine aldehyde dehydrogenase 1	AT1G74920	F4HXD2	0.037	0.26
Chalcone synthase	AT5G13930	P13114	0.039	0.26
**Hormone metabolism**				
Delta(24)-sterol reductase	AT3G19820	Q39085	0.037	0.31
**Co-factor and vitamin metabolism**				
Nicotinate-nucleotide pyrophosphorylase (carboxylating)	AT2G01350	F41NA0	0.015	0.10
**Cell wall synthesis**				
UDP-glucuronic acid decarboxylase 3	AT5G59290	F4KHU8	0.029	2.93
**Tetrapyrrole synthesis**				
Protoporphyrinogen oxidase 2	AT5G14220	Q8S9J1-2	0.003	0.19
**Abiotic stress**				
Endoplasmin homolog	AT4G24190	F4JQ55	0.031	3.16
DnaJ protein ERDJ38	AT3G62600	Q9LZK5	0.002	6.67
Probable methyltransferase PMt24	AT1G29470	Q6NPR7	0.004	5.51
Germin-like protein subfamily T member 1	AT1G18970	P92995	0.031	3.55
**Redox response**				
Protein disulfide isomerase-like 1-2	AT1G21750	F4HZN9	0.036	4.41
Protein disulfide-isomerase	AT1G77510	Q9SRG3	0.002	8.12
Protein disulfide isomerase-like 1-6	AT3G16110	Q66GQ3	0.025	0.21
Thioredoxin reductase 2	AT2G17420	Q39242	0.013	0.28
**Nucleotide metabolism**				
Nudix hydrolase 3	AT1G79690	Q8L831	0.018	0.26
**RNA processing**				
Polyadenylate-binding protein 2	AT4G34110	P42731	0.048	0.39
Polyadenylate-binding protein 4	AT2G23350	O22173	0.006	0.18
Reactive intermediate deaminase A	AT3G20390	Q94JQ4	0.002	11.07
**Protein synthesis**				
30S ribosomal protein S3	ATCG00800	P56798	0.043	2.82
40S ribosomal protein S3-3	AT5G35530	Q9FJA6	0.004	7.52
Elongation factor Tu (mitochondrial)	AT4G02930	Q9ZT91	0.034	3.43
Elongation factor Tu (chloroplastic)	AT4G20360	P17745	0.026	5.32
**Protein degradation**				
26S proteasome non-ATPase regulatory subunit 14 homolog	AT5G23540	Q9LT08	0.012	0.27
Proteasome subunit beta type-4	AT1G56450	Q7DLR9	0.035	0.27
**Protein targeting**				
Nuclear pore complex protein NUP155	AT1G14850	F4HXV6	0.033	2.99
ADP-ribosylation factor 2-A	AT3G62290	Q9M1P5	0.031	4.87
**Signaling**				
Rho GDP-dissociation inhibitor 1	AT3G07880	Q9SFC6	0.021	5.59
Dynamin-related protein 1A	AT5G42080	P42697	0.003	5.79
GTP-binding nuclear protein Ran-1	AT5G20010	P41916	0.020	4.92
14-3-3-like protein GF14 chi	AT4G09000	P42643	0.032	0.22
**Cell vesicle transport**				
Tubulin beta 6-chain (Cell organization)	AT5G12250	P29514	0.020	0.28
AP-4 complex subunit epsilon	AT1G31730	Q8L7A9	0.030	3.63
Coatomer subunit beta-1	AT4G31480	Q95V21	0.007	8.98
Golgin candidate 5	AT1G79830	F4HQB9	0.015	5.49
COG complex component-related protein	AT5G51430	Q9FGN0	0.023	6.23
**Transport**				
V-type proton ATPase subunit E3	AT1G64200	P0CAN7	0.044	2.89
ATPase 2, plasma membrane-type	AT4G30190	P19456	0.046	3.26
Mitochondrial dicarboxylate/tricarboxylate transporter DTC	AT5G19760	Q9C5M0	0.010	3.54
ABC transporter A family member 2	AT3G47730	Q84K47	0.010	3.82
ABC transporter F family member 1	AT5G60790	Q9FJH5	0.009	0.15
**Miscellaneous**				
AT3g23600/MDB19_9	ATG23600	Q9LUG8	0.012	4.83
Dolichyl-diphosphooligosaccharide-protein glycosyltransferase 48 kDa subunit	AT5G66680	Q944K2	0.009	0.17
Methylesterase 3	AT2G23610	O80477	0.0369	2.93
Glutathione S-transferase L3	AT5G02790	Q9LZO6	0.0218	3.61
Peroxidase 34	AT3G49120	Q9SMU8	0.0070	0.16
Peroxidase 30	AT3G21770	Q9LSY7	0.0165	4.46
AT4g13180/F17N18-70	AT4G13180	Q9SVQ9	0.0322	0.22
**Not assigned ontology**				
NADH dehydrogenase (ubiquinone) iron-sulfur protein 2	ATMG00510	P93306	0.0167	1.78
tRNA (guanine-N(7)-)-methyltransferase non-catalytic subunit	AT1G03110	Q93WD7	0.0376	0.31
Pheromone receptor, putative (AR401)	AT1G66680	Q9C9M1	0.0143	4.38
WD40 domain-containing protein	AT5G24710	F4K1H8	0.0265	0.29
Protein EMBRYO DEFECTIVE 2734	AT5G19820	Q93V68	0.0208	0.20
Calcium-dependent lipid-binding family protein	AT1G48090	F4HWS2	0.0156	0.29
Metal-dependent protein hydrolase	AT5G41970	F4K000	0.0349	3.09

**Table 3:** Individual glucosinolate content (ng mg^−1^ FW) in roots of *A. thaliana* plants grown with nitrate or ammonium as sole N source.

Aliphatic Glucosinolates	Nitrate	Ammonium
Glucoraphanin (4MSOB)	25.61 ± 4.13	**64.12 ± 8.76**
Glucoalyssin (5MSOP)	3.59 ± 0.32	**6.04 ± 0.52**
Glucoiberin (3MSOP)	1.88 ± 0.27	**4.50 ± 0.55**
Glucoerucin (4MTB)	2.20 ± 0.52	**4.25 ± 0.38**
Glucoberteroin (5MTP)	0.87 ± 0.08	**1.50 ± 0.08**
Glucoibarin (7MSOH)	54.17 ± 4.76	44.56 ± 3.01
Glucohirsutin (8MSOO)	610.00 ± 57.62	**452.58 ± 33.60**
C6-aliphatic GLS A (C_13_H_24_NO_9_S_2_)	0.57 ± 0.07	0.80 ± 0.10
7-Methylthioheptyl-GS (C_15_H_28_NO_9_S_3_)	125.12 ± 11.28	**92.59 ± 4.57**
8-Methylthiooctyl-GS (C_16_H_31_NO_9_S_3_)	1090.72 ± 58.47	**786.08 ± 33.54**
**Total Aliphatic**	1914.67 ± 125.02	**1457.04 ± 69.85**
**Indolic Glucosinolates**	**Nitrate**	**Ammonium**
Glucobrassicin (I3M)	96.97 ± 8.25	97.54 ± 4.64
Neoglucobrassicin (IMOI3M)	268.83 ± 25.97	214.38 ± 16.71
Hydroxyglucobrassicin (4OHI3M)	12.39 ± 1.00	11.73 ± 0.37
Methoxyglucobrassicin (4MOI3M)	21.90 ± 2.62	20.87 ± 2.58
**Total Indolic**	400.10 ± 36.42	344.52 ± 22.24
**Total Glucosinolates**	2337.88 ± 158.74	**1818.12 ± 90.34**

Values represent mean ± SE (*n* = 4). Significant differences among treatments are highlighted in bold text (Student *t*-test, *p* < 0.05).

## Supplementary Materials

Supplementary materials can be found at http://www.mdpi.com/1422-0067/20/4/814/s1.

## Abbreviations

2-OG	2-Oxoglutarate
AACT	Acetoacetyl-CoA thiolase
ACCT	Acetyl-CoA acetyltransferase
ACL	ATP-citrate lyase
AMT	Ammonium transporter
AOX	Alternative oxidase
ATP	Adenosine triphosphate
BR	Brassinosteroid
CHS	Chalcone synthase
CoA	Coenzyme A
CS	Citrate synthase
DMAP	Dimethylallyl phosphate
DMAPP	Dimethylallyl diphosphate
FDR	False discovery rate
FPP	Farnesyl diphosphate
FW	Fresh weight
GAD	Glutamate decarboxylase
GABA	Gamma-aminobutyric acid
GDH	Glutamate dehydrogenase
GLS	Glucosinolate(s)
GO	Gene ontology
GOGAT	Glutamate synthase
GS	Glutamine synthetase
ICDH	Isocitrate dehydrogenase
IP	Isopentenyl phosphate
IPP	Isopentenyl diphosphate
iTRAQ	Isobaric tags for relative and absolute quantitation
MDH	Malate dehydrogenase
ME	Malic enzyme
MVA	Mevalonate
MVAP	Phosphomevalonate
NAD(H)	Nicotinamide adenine dinucleotide (reduced)
NADP(H)	Nicotinamide adenine dinucleotide phosphate (reduced)
NUDIX 3	Nudix hydrolase and a dipeptidyl peptidase III
OAA	Oxaloacetate
PEP	Phosphoenolpyruvate
PEPCK	Phosphoenolpyruvate carboxykinase
PEPC	Phosphoenolpyruvate carboxylase
PK	Pyruvate kinase
TCA	Tricarboxylic acid
